# Incorporating basic periodontal screening into antenatal care services provided in Rwanda: A policy brief

**DOI:** 10.12688/f1000research.152760.2

**Published:** 2024-08-05

**Authors:** Peace Uwambaye, Kehinde Kazeem Kanmodi

**Affiliations:** 1 Preventive and Community Dentistry, University of Rwanda, Kigali city, Rwanda; 2 School of Health and Life Sciences, Teeside University, London, Middlesbrough, UK

**Keywords:** Antenatal care, periodontitis, preterm birth, preterm deliveries, policy brief, Rwanda

## Abstract

**Background:**

Preterm birth, which is child delivery before 37 weeks of pregnancy, is the primary cause of perinatal mortality worldwide. Preterm birth remains a major public health challenge in Rwanda, affecting approximately 13.8% of birth. The World Health Organization estimates that 15 million babies are born prematurely each year. While the association between periodontitis and preterm deliveries is increasingly recognized, little is known about this link in Rwanda. This policy brief aims to bridge this knowledge gap by presenting the findings of a recent study investigating the prevalence of periodontitis among pregnant women in Rwanda and its association with preterm deliveries. This policy brief also aims to inform and guide decision making towards incorporating periodontal screening into the antenatal care package in Rwanda. This has the potential to improve pregnancy outcomes and contribute to improved oral health in the future.

**Policy outcome and Implications:**

Contemporary evidence has shown a six-fold increased risk of preterm delivery for women with periodontitis in Rwanda, with a concerningly high prevalence (60%) among pregnant women. Additionally, nurses working in antenatal clinics displayed insufficient knowledge about gum disease.

**Actionable recommendations:**

The adoption of basic periodontal screening within existing antenatal care packages is recommended. This, coupled with nurse training and public awareness campaigns, can empower women and healthcare professionals to prioritize oral health for better pregnancy outcomes.

**Conclusions:**

Integrating periodontal screening into antenatal care has the potential to significantly reduce preterm deliveries and contribute to a healthier future generation in Rwanda.

## Background

Preterm birth, which is child delivery before 37 weeks of pregnancy, is the primary cause of perinatal mortality worldwide and a risk factor for preterm delivery. It remains a major public health concern in Rwanda, affecting approximately 13.8% of birth. ^1^^–^^3^ The World Health Organization estimates that 15 million babies are born prematurely each year. ^1^ Periodontitis is a chronic inflammatory disease of the gums that can lead to tooth loss. It is one of the most common chronic diseases in the world, affecting nearly half of all adults. ^4^

While the association between periodontitis and preterm deliveries is increasingly recognized, little is known about this link in Rwanda. This policy brief aims to bridge this knowledge gap by presenting the findings of a recent study investigating the prevalence of periodontitis among pregnant women in Rwanda and its association with preterm deliveries. This policy brief also aims to inform and guide decision making towards incorporating periodontal screening into the antenatal care package in Rwanda. This has the potential to improve pregnancy outcomes and contribute to improved oral health in the future.

## Policy outcomes and implications

Different studies have suggested a complex link between periodontitis and preterm birth. Chronic inflammation in the gums associated with periodontitis releases inflammatory mediators into the blood stream and this potentially triggers early labor through various biomedical pathways. ^5^^–^^8^ Also, oral bacteria from infected gums can reach the placenta and amniotic fluid, triggering an inflammatory response in the uterus and inducing preterm labor. ^9^^,^^10^ Also, periodontitis can impair nutrient absorption, impacting fetal growth and development which may contribute to preterm birth. ^11^ Periodontal diseases occur as result of dysbiosis in bacterial biofilm inhabiting the sulcus, below the gingival margin. Periodontal diseases are inclusive of the main diagnostic categories of periodontitis and gingivitis. ^7^^,^^8^^,^^12^

Existing research from Rwanda, like some other African countries, demonstrates a significant association between periodontitis and preterm birth. A study in Kenya reported that women with periodontitis have 2 times risk of giving birth to preterm babies than women without periodontitis. ^13^ In Ethiopia, a study reported 2-4 times the odds of giving birth to preterm babies in women with periodontitis ^14^ while in Rwanda is six-fold risk of preterm in women with periodontitis than those with no periodontitis. ^15^

The above findings show that Rwandan pregnant women with periodontitis have higher risks of preterm deliveries compared to those in neighboring countries. ^13^^–^^15^ Further, research has also shown that nurses and midwives in Rwanda had insufficient knowledge on periodontitis. ^16^ Therefore, there is a need for a tool to nurses and midwives in Rwanda in the detection of periodontal diseases among pregnant women during antenatal care consultations.

Periodontal screening is not currently part of the antenatal care package in Rwanda, despite the increasing prevalence of periodontitis among pregnant women in Rwanda. Research evidence has shown the strong association between periodontitis and preterm birth. ^15^ Several studies confirm that the treatment of periodontitis during pregnancy reduces the risk of preterm delivery. ^17^^–^^21^

Antenatal care consultations constitute an important opportunity to perform screening for periodontitis during pregnancy in the context of Rwanda. Firstly, antenatal care consultations are mandatory for all pregnant women. Secondly, the uptake is high (98%) and a greater number of pregnant women meet the required number of antenatal care visits. ^22^

The increasing uptake of antenatal care services in Rwanda is supported by more comprehensive medical insurance schemes (e.g “Mutuel de Sante” that covers 85% of the cost). Community health workers (CHWs) identify all pregnant women in their villages and follow them up until delivery. CHWs ensure that these pregnant women attend antenatal care.

Oral diseases, including periodontitis, are neglected and it is rare for a woman to go for dental check-ups unless when in pain. Antenatal care is an important platform that offers the opportunity to bring awareness on periodontitis during pregnancy. Periodontitis screening during antenatal care consultations will facilitate the access of all pregnant women to oral health.

The development of a periodontal screening tool that can be utilized and integrated in an antenatal care package would therefore be desirable and cost-effective. Thus, this policy brief calls for the development of a screening tool as well as the sustainable implementation of such a tool. If a robust screening tool is successfully developed, then future studies may be conducted to explore the management of periodontitis during pregnancy and its effect on preterm delivery in Rwanda.

## Actionable recommendations

To address the potential link between periodontitis and preterm birth in Rwanda, multiple recommendations are proposed. Some of these recommendations were raised during the policy engagement workshop that was held and led by the lead author of this policy brief, in October 2023, where there was a presentation of the study findings to different policy makers and partners that included professional associations, representatives from hospitals and representatives from Rwanda Ministry of Health through the Rwanda Biomedical Center. These recommendations are below:

***The incorporation of Periodontal Screening in Antenatal Care***: There is a need to integrate a basic periodontal screening into the existing Rwandan ANC package. This screening can be performed by trained nurses and could involve visual inspection and probing to identify signs of gum inflammation and potential periodontitis. This can be done by developing clear protocols and guidelines for nurses on how to conduct the screening, interpret results, and refer women with suspected periodontitis to dentists for further evaluation and treatment.

***Capacity Building for Nurses:*** There is need to implement training programs to equip nurses working in antenatal clinics with the knowledge and skills to effectively: conduct basic periodontal screenings, educate pregnant women about oral hygiene practices and the link between gum health and pregnancy outcomes and advise women on appropriate referrals for periodontal treatment when necessary.

***Public Awareness Campaigns:*** There is a need to develop and disseminate public awareness campaigns to educate women of childbearing age about periodontal disease, its risks during pregnancy, and the importance of maintaining good oral hygiene.

## Conclusion

While the causal relationship between periodontitis and preterm birth requires further investigation, the existing evidence suggests a potentially significant link. By integrating oral health into maternal care, promoting oral hygiene practices, and conducting further research, we can take crucial steps to address this issue and improve maternal and child health outcomes in Rwanda. Policymakers, healthcare professionals, and community leaders in Rwanda should recognize the potential impact of periodontitis on preterm birth. By implementing the recommended actions and prioritizing oral health promotion, we can work together to ensure mothers and babies have a healthy start in life.

## Author contributions

Both authors (PU and KK) contributed to writing of the original draft, reviewing, editing and conceptualization of the manuscript.

## Ethics and consent

Ethical approval and consent were not required.

## Data Availability

No data are associated with this article.
