# Supplementary material for: Incorporating basic periodontal screening into antenatal care services provided in Rwanda: A policy brief
Source: F1000Res. 2024 Aug 5;13:647. Originally published 2024 Jun 17. [Version 2] doi: 10.12688/f1000research.152760.2 (PMC11347909; doi:10.12688/f1000research.152760.2)
Supplement: Peer review history

# Reviewer report: Reviewer response for version 2

**DOI:** 10.5256/f1000research.169859.r310988

**Published:** 2024-08-26

**Authors:** Jaume Miranda-Rius

**Affiliations:** 1 Sant Joan de Déu Hospital, Institut de Recerca Sant Joan de Déu (IRSJD), University of Barcelona, Barcelona, Spain; 2 Faculty of Medicine and Health Sciences, University of Barcelona, Barcelona, Catalonia, Spain

The authors have addressed consistently all reviewers' comments.

Does the paper provide a comprehensive overview of the policy and the context of its implementation in a way which is accessible to a general reader?

Yes

Is the discussion on the implications clearly and accurately presented and does it cite the current literature?

No

Are the recommendations made clear, balanced, and justified on the basis of the presented arguments?

Yes

Reviewer Expertise:

Periodontology and Periodontal Medicine

I confirm that I have read this submission and believe that I have an appropriate level of expertise to confirm that it is of an acceptable scientific standard.

# Reviewer report: Reviewer response for version 2

**DOI:** 10.5256/f1000research.169859.r310987

**Published:** 2024-08-07

**Authors:** Juliana Velosa-Porras

**Affiliations:** 1 Pontificia Universidad Javeriana, Bogotá, Colombia

Dear Authors. My suggestions were taken into account and added to the text.

Does the paper provide a comprehensive overview of the policy and the context of its implementation in a way which is accessible to a general reader?

Yes

Is the discussion on the implications clearly and accurately presented and does it cite the current literature?

No

Are the recommendations made clear, balanced, and justified on the basis of the presented arguments?

Yes

Reviewer Expertise:

Clinical Epidemiology, Epidemiology, Periodontics, Periodontal disease and its relationship to systemic diseases, pregnancy and oral health

I confirm that I have read this submission and believe that I have an appropriate level of expertise to confirm that it is of an acceptable scientific standard.

# Reviewer report: Reviewer response for version 1

**DOI:** 10.5256/f1000research.167558.r298808

**Published:** 2024-07-22

**Authors:** Juliana Velosa-Porras

**Affiliations:** 1 Pontificia Universidad Javeriana, Bogotá, Colombia

Dear Authors,

 It was a pleasure for me to read your manuscript. The research topic is of interest, countries with limited resources should incorporate these strategies to promote oral health and prevent diseases such as preterm birth. As the first point of contact with patients, this manuscript shows the fundamental role of nurses and midwives in the prevention of oral diseases.

 Minor revision

 I suggest this two references:

- Pockpa ZAD, et. al., 2022 (Ref 1)
- Muwazi L, et. al., 2014 (Ref 2)

**Background**

 I suggest that sentences 1 and 6 in the first paragraph could be synthesized – the information is the same.

**Policy outcomes and implications**

 Please check the wording in the last sentence of the first paragraph.

 I suggest that the second paragraph focus on the association of periodontitis and preterm birth. In the third paragraph the information on the association of periodontitis and preterm birth is unnecessary.

 Third paragraph, line 3 – " There is a need fora tool to support the nurses/midwives to detect periodontal diseases among pregnant women during antenatal care consultations.…" Please check the wording.

**Actionable recommendations**

 First paragraph, line 2 – " ….. the lead author of this policy brief, in October 2024 where there…..” Please check the wording. Please check this date, since it has not yet passed, I believe it was a typing error.

Does the paper provide a comprehensive overview of the policy and the context of its implementation in a way which is accessible to a general reader?

Yes

Is the discussion on the implications clearly and accurately presented and does it cite the current literature?

No

Are the recommendations made clear, balanced, and justified on the basis of the presented arguments?

Yes

Reviewer Expertise:

Clinical Epidemiology, Epidemiology, Periodontics, Periodontal disease and its relationship to systemic diseases, pregnancy and oral health

I confirm that I have read this submission and believe that I have an appropriate level of expertise to confirm that it is of an acceptable scientific standard.

## References

[1] : Association Between Periodontitis and Preterm Birth in a Cohort of Pregnant Women in Ivory Coast. *Oral Health Prev Dent* .2022;20(1) : 10.3290/j.ohpd.b3464893 363-368 10.3290/j.ohpd.b3464893 36259439 PMC11640969

[2] : Periodontal conditions, low birth weight and preterm birth among postpartum mothers in two tertiary health facilities in Uganda. *BMC Oral Health* .2014;14: 10.1186/1472-6831-14-42 42 10.1186/1472-6831-14-42 24773772 PMC4022576

# Response

**Published:** 2024-07-30

**Authors:** Peace UWAMBAYE

**Affiliations:** Preventive and Community Dent, University of Rwanda, Kigali, Rwanda

**REVIEWER 2**

 Dear Authors,

 It was a pleasure for me to read your manuscript. The research topic is of interest, countries with limited resources should incorporate these strategies to promote oral health and prevent diseases such as preterm birth. As the first point of contact with patients, this manuscript shows the fundamental role of nurses and midwives in the prevention of oral diseases.

 Minor revision

 I suggest this two references:

- Pockpa ZAD, et. al., 2022 (Ref 1)
- Muwazi L, et. al., 2014 (Ref 2)

**Response: **Thank you. We have now cited these two references in the revised manuscript.

**Reviewer 2- Background:**

 I suggest that sentences 1 and 6 in the first paragraph could be synthesized – the information is the same.

**Response: **Thank you. Both sentences have now been synthesized.

**Reviewer 2- Policy outcomes and implications:**

 Please check the wording in the last sentence of the first paragraph.

**Response: **Thank you. This sentence has now been revised.

**Reviewer 2: **I suggest that the second paragraph focus on the association of periodontitis and preterm birth. In the third paragraph the information on the association of periodontitis and preterm birth is unnecessary.

**Response: **Thank you. This sentence, and paragraph, has now been revised.

**Reviewer 2: **Third paragraph, line 3 – " There is a need fora tool to support the nurses/midwives to detect periodontal diseases among pregnant women during antenatal care consultations.…" Please check the wording.

**Response: **Thank you. This sentence has now been revised.

**Reviewer 2- Actionable recommendations:**

 First paragraph, line 2 – " ….. the lead author of this policy brief, in October 2024 where there…..” Please check the wording. Please check this date, since it has not yet passed, I believe it was a typing error.

**Response: **Thank you. This sentence has now been revised.

**Reviewer 2- References:**

 1. Pockpa ZAD, Soueidan A, Koffi-Coulibaly NT, Mobio GS, et al.: Association Between Periodontitis and Preterm Birth in a Cohort of Pregnant Women in Ivory Coast. *Oral Health Prev Dent*. 2022; **20** (1): 363-368 PubMed Abstract | Publisher Full Text

 2. Muwazi L, Rwenyonyi CM, Nkamba M, Kutesa A, et al.: Periodontal conditions, low birth weight and preterm birth among postpartum mothers in two tertiary health facilities in Uganda. *BMC Oral Health*. 2014; **14**: 42 PubMed Abstract | Publisher Full Text

**Response: **Thank you. We have now cited these two references in the revised manuscript.

# Reviewer report: Reviewer response for version 1

**DOI:** 10.5256/f1000research.167558.r294152

**Published:** 2024-07-03

**Authors:** Jaume Miranda-Rius

**Affiliations:** 1 Sant Joan de Déu Hospital, Institut de Recerca Sant Joan de Déu (IRSJD), University of Barcelona, Barcelona, Spain; 2 Faculty of Medicine and Health Sciences, University of Barcelona, Barcelona, Catalonia, Spain

Dear Authors,

 I enjoyed reading this manuscript. Especially in countries where there is a shortage of dental surgeons it is important to involve nurses in oral health education. Maternity is important in women's lives so midwives need to acquire knowledge about periodontitis and its possible repercussions on the foeto-placental unit. 

 Perhaps I could suggest to the authors to add these three references as they are studies also carried out in East Africa. 

 Gesase N, et. al., 2018 (Ref 1)

 Miranda-Rius J, et. al., 2023 (Ref 2)

 Coleman M, et. al., 2019 (Ref 3)

 Minor Revision:

 COMMENTS TO THE DOCUMENT

 INCORPORATING BASIC SCREENING INTO ANTENATAL

 CARE SERVICES PROVIDED IN RWANDA: A POLICY BRIEF

 Peace Uwambaye, Kehinde Kazeem Kanmodi (2024)

 This is a brief policy brief on the issue of preterm births in Rwanda. It has been shown that the rate of these births is very high. Simultaneously, several studies have found periodontitis to be a risk factor for preterm birth (<37 weeks). The aim of this study is to assess the prevalence of periodontitis in the country and to recommend a social policy of periodontitis control in pregnant women: diagnosis and prenatal care to reduce it and decrease the incidence of preterm births.

 Some objectives to be commented on:

 --In section "Policy outcomes and implications", 4th paragraph, last line, there is a reference to some studies but the citations of these studies are not given. They should be referenced.

 -- It is said that periodontal screening should be carried out on pregnant women to find out their periodontal situation, and from there to apply statistics, records and treatment; but it is not explained how this is to be carried out. No action plan is specified. It is only recommended to implement actions to prioritise oral health and to establish joint boundaries between specialties. Therefore, it will be good to start from this document a prospective cohort study design, in order to present guidelines to the Ministry of Health.

Does the paper provide a comprehensive overview of the policy and the context of its implementation in a way which is accessible to a general reader?

Yes

Is the discussion on the implications clearly and accurately presented and does it cite the current literature?

No

Are the recommendations made clear, balanced, and justified on the basis of the presented arguments?

Yes

Reviewer Expertise:

Periodontology and Periodontal Medicine

I confirm that I have read this submission and believe that I have an appropriate level of expertise to confirm that it is of an acceptable scientific standard, however I have significant reservations, as outlined above.

## References

[1] : The association between periodontal disease and adverse pregnancy outcomes in Northern Tanzania: a cross-sectional study. *Afr Health Sci* .2018;18(3) : 10.4314/ahs.v18i3.18 601-611 10.4314/ahs.v18i3.18 30602993 PMC6307003

[2] : Microbial profile of placentas from Tanzanian mothers with adverse pregnancy outcomes and periodontitis. *Oral Dis* .2023;29(2) : 10.1111/odi.13962 772-785 10.1111/odi.13962 34255399

[3] : Inhibition of the Chemokine Response Prevents Preterm Labor but Not an Invasive Infection in a Nonhuman Primate Model. *https://link.springer.com/article/10.1177/1933719119834079?utm_source* .2019; 10.1177/1933719119834079 10.1177/1933719119834079

# Response

**Published:** 2024-07-30

**Authors:** Peace UWAMBAYE

**Affiliations:** Preventive and Community Dent, University of Rwanda, Kigali, Rwanda

**REVIEWER 1**

 Dear Authors,

 I enjoyed reading this manuscript. Especially in countries where there is a shortage of dental surgeons it is important to involve nurses in oral health education. Maternity is important in women's lives so midwives need to acquire knowledge about periodontitis and its possible repercussions on the foeto-placental unit. 

**Response: **Thank you.

**REVIEWER 1: **Perhaps I could suggest to the authors to add these three references as they are studies also carried out in East Africa. 

 Gesase N, et. al., 2018 (Ref 1)

 Miranda-Rius J, et. al., 2023 (Ref 2)

 Coleman M, et. al., 2019 (Ref 3)

**Response: **Thank you. We have now cited these three references in the revised manuscript.

**REVIEWER 1: **This is a brief policy brief on the issue of preterm births in Rwanda. It has been shown that the rate of these births is very high. Simultaneously, several studies have found periodontitis to be a risk factor for preterm birth (<37 weeks). The aim of this study is to assess the prevalence of periodontitis in the country and to recommend a social policy of periodontitis control in pregnant women: diagnosis and prenatal care to reduce it and decrease the incidence of preterm births.

 Some objectives to be commented on:

 --In section "Policy outcomes and implications", 4th paragraph, last line, there is a reference to some studies but the citations of these studies are not given. They should be referenced.

**Response: **Thank you. We have now provided references to support this sentence. We cited the three references you recommended as well as the two references recommended by Reviewer 2.

**Reviewer 1:** It is said that periodontal screening should be carried out on pregnant women to find out their periodontal situation, and from there to apply statistics, records and treatment; but it is not explained how this is to be carried out. No action plan is specified. It is only recommended to implement actions to prioritise oral health and to establish joint boundaries between specialties. Therefore, it will be good to start from this document a prospective cohort study design, in order to present guidelines to the Ministry of Health.

**Response: **Thank you. We would like to avoid being too specific by saying that a prospective cohort study design should be conducted in our recommendations, because all research investigations should be welcome by the government on this area. However, this we will put at the back of our mind. We will be very glad to pursue a research project on that and we are very open to collaborative opportunities.

**Reviewer 1- References:**

 1. Gesase N, Miranda-Rius J, Brunet-Llobet L, Lahor-Soler E, et al.: The association between periodontal disease and adverse pregnancy outcomes in Northern Tanzania: a cross-sectional study. *Afr Health Sci*. 2018; **18** (3): 601-611 PubMed Abstract | Publisher Full Text

 2. Miranda-Rius J, Brunet-Llobet L, Blanc V, Álvarez G, et al.: Microbial profile of placentas from Tanzanian mothers with adverse pregnancy outcomes and periodontitis. *Oral Dis*. 2023; **29** (2): 772-785 PubMed Abstract | Publisher Full Text

 3. M, Coleman A, Orvis S, Merillat J, et al.: Inhibition of the Chemokine Response Prevents Preterm Labor but Not an Invasive Infection in a Nonhuman Primate Model. *https://link.springer.com/article/10.1177/1933719119834079?utm_source*. 2019. 

**Response: **Thank you. We have now cited these three references in the revised manuscript.
